# Acoustic force mapping in a hybrid acoustic-optical micromanipulation device supporting high resolution optical imaging†
†Electronic supplementary information (ESI) available: Additional information about 1D model calculations for a piezoelectric transducer. See DOI: 10.1039/c6lc00182c



**DOI:** 10.1039/c6lc00182c

**Published:** 2016-03-17

**Authors:** Gregor Thalhammer, Craig McDougall, Michael Peter MacDonald, Monika Ritsch-Marte

**Affiliations:** a Division of Biomedical Physics , Medical University Innsbruck , Müllerstraße 44 , 6020 Innsbruck , Austria . Email: gregor.thalhammer@i-med.ac.at ; Tel: +43 512 9003 70875; b Division of Cancer Research , School of Medicine , University of Dundee , Dundee , UK; c Physics, School of Science and Engineering , University of Dundee , Dundee , UK

## Abstract

Many applications in the life-sciences demand non-contact manipulation tools for forceful but nevertheless delicate handling of various types of sample. Moreover, the system should support high-resolution optical imaging. Here we present a hybrid acoustic/optical manipulation system which utilizes a transparent transducer, making it compatible with high-NA imaging in a microfluidic environment. The powerful acoustic trapping within a layered resonator, which is suitable for highly parallel particle handling, is complemented by the flexibility and selectivity of holographic optical tweezers, with the specimens being under high quality optical monitoring at all times. The dual acoustic/optical nature of the system lends itself to optically measure the exact acoustic force map, by means of direct force measurements on an optically trapped particle. For applications with (ultra-)high demand on the precision of the force measurements, the position of the objective used for the high-NA imaging may have significant influence on the acoustic force map in the probe chamber. We have characterized this influence experimentally and the findings were confirmed by model simulations. We show that it is possible to design the chamber and to choose the operating point in such a way as to avoid perturbations due to the objective lens. Moreover, we found that measuring the electrical impedance of the transducer provides an easy indicator for the acoustic resonances.

## Abstract 2

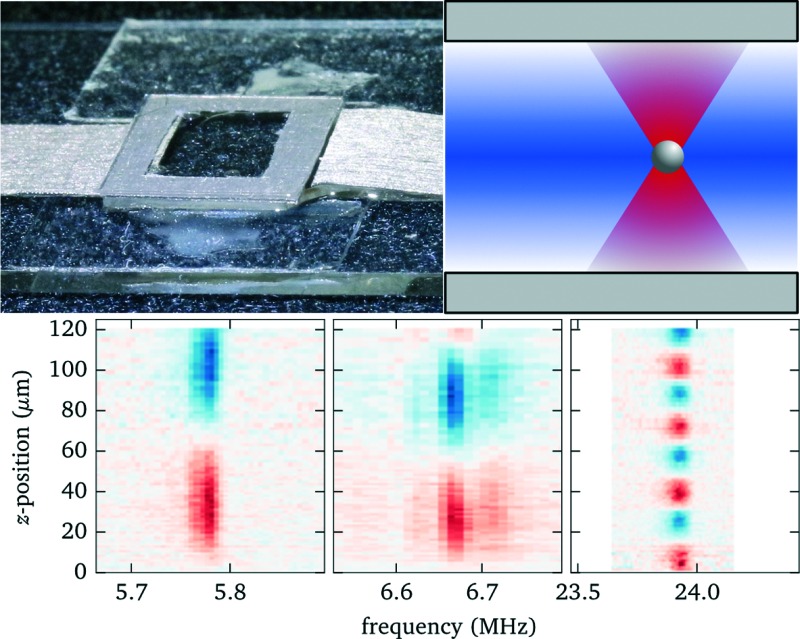
We demonstrate combined acoustic-optical trapping with transparent piezoelectric transducers supporting high-resolution imaging and acoustic force mapping.

## 1 Introduction

Acoustic forces have the ability to simultaneously trap or manipulate many (thousands) of small particles, while providing sufficiently strong forces at low power densities, thus hardly affecting the behaviour or viability of biological samples. This is beneficial for the scaling and parallelization in life science applications.^1,2^ On the other hand, optical tweezers are a preeminent tool for the contactless manipulation of individual particles with high precision and provide a way to apply and measure forces on the micro-scale.^3,4^ Furthermore, high quality optical microscopy is one of the primary research tools in this field. When combined in a single setup, acoustic and optical trapping complement each other and provide new possibilities for particle manipulation and inspection, which cannot be realized with a single technique alone. One specific example, as demonstrated in this work, is the trapping, manipulation and imaging of larger particles (*Lycopodium* spores).

### Requirements for scalable acoustic trapping

For applications employing acoustic forces the design and characterization of the setup is often a non-trivial task. To achieve significant forces it is common practice to exploit resonances. Particles are driven to the (pressure) nodes of a resonant standing wave, the shape of which depends on the geometrical design and material properties of the device. Different acoustic resonator designs, compatible with micro-fluidics, have been realized, which can be roughly divided into two groups:^5^ in transverse resonators the acoustic wave travels in the transverse direction relative to the observation direction. Conversely, in a layered resonator, which typically consists of a planar fluid layer enclosed by glass layers, acting as reflectors, the acoustic wave travels normal to the observation plane. Each variant has its distinct value: transverse resonators typically employ more complex patterns and provide more possibilities to tailor the force landscape. Therefore they are more appropriate if one is interested in dextrous particle handling by acoustic forces alone. Additionally, in transverse resonators the observation of the effect of acoustic forces is easier, since they act normal to the observation direction, and the forces can be measured, *e.g.*, by particle image velocimetry (PIV),^6–8^ with optical tweezers,^9–11^ or deduced from interferometric measurements of the sound pressure.^12^

On the other hand, layered resonators, which we address in this work, are easy to realize and provide robust and efficient operation. They are easy to scale up by increasing the area, and thus better suited for applications where massively parallel handling is desired. Furthermore, particles are confined within a plane parallel to the layers, which enables convenient simultaneous imaging of all particles. However, as force measurements in layered resonators require more involved methods (such as 3D particle tracking by stereomicroscopy^13^), to our best knowledge no detailed, quantitative experimental characterization of the acoustic forces in planar devices has previously been published.

### Requirements for imaging and optical manipulation

For high quality imaging it is mandatory to use a high-NA immersion objective lens, which is needed to achieve high resolution and/or to collect as much light as possible, *e.g.* for fluorescence microscopy. Proper illumination is also crucial. Similarly, single beam optical trapping requires focussing of the trapping light with a high-NA lens, while sensitive force measurements rely on recollecting the scattered trapping light.

### Challenges in combining acoustic and optical trapping

When combining acoustic and optical manipulation all of the aforementioned requirements need to be taken into account. In particular, as tiny changes to the device can influence the acoustic resonance frequencies and mode shapes due to the often favored large quality factors^14,15^ (ratio of the stored to dissipated energy) in the order of 100–1000,^15–17^ special attention is required when bringing a high-NA objective immersion lens in close contact with the acoustic trapping device.^18^ Ideally, the acoustic resonances within the fluid layer should not be affected by adding more layers.

### Main achievements

As a main contribution of this work we present the combination of an optically transparent layered acoustic resonator with optical tweezers and combine it with a robust method to optically measure the axial force acting on a trapped particle. Importantly, our setup also permits high-NA imaging. As a first application of the approach we quantitatively characterize the (acoustic) properties of the probe chamber, such as resonance strength, resonance frequencies, and force profiles, in particular in the axial direction. Our results show that the perturbations in the acoustic resonance due to the close contact of the objective with the probe chamber follow simple rules, allowing for straightforward strategies to tackle arising issues. In particular we show how to avoid situations where the acoustic resonance within the fluid layer is severely affected.

In order to realize our work we employ several recent achievements which, in combination with established methods, were crucial to performing our measurements:

• We implement holographic optical trapping (HOT). Utilizing a spatial light modulator (SLM) to shape the trapping beam, we are able to translate the optical trap position along the axial direction over the full extent of the probe chamber, without moving the objective lens. This allows the measurement of axial force profiles without modifying the acoustic properties.

• In order to characterize the acoustic forces with the optical tweezers we use the direct force measurement method,^19,20^ which relies on analyzing the angular intensity distribution of the transmitted trapping light, directly revealing the amount of momentum transferred from the trapping beam to the particle. This has been proven to be a robust method that is essentially calibration free, enabling productive and fast measurements.

• To implement the direct force measurement method in the combined setup it is essential to use a transparent acoustic transducer, in this case made out of LiNbO_3_ with transparent conductive indium tin oxide (ITO) coating as electrodes, so that the transmitted trapping light can be collected.^21^

• Additional to the optical measurement of the acoustic forces within the fluid layer we perform measurements of the electrical response (impedance) of the acoustic transducer. This provides complementary information about all the acoustic resonances (in any layer) within the setup, in particular the resonance frequencies.

• A comparison of the measured data with theoretical model calculations allows us to determine the value of experimental parameters, such as layer thicknesses or the speed of sound in the materials, which are not precisely enough known beforehand. Based on the refined model, which shows a good agreement between experiment and simulations, we are able to predict the properties (resonance frequencies, force profiles and strength) of modified setups.

## 2 Experimental setup

### 2.1 Probe chamber design for simultaneous acoustic and optical manipulation

**Fig. 1:**
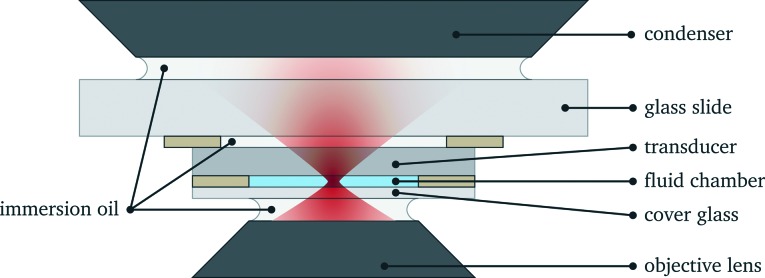
Schematic cross section through mounted probe chamber (not to scale).

**Fig. 2:**
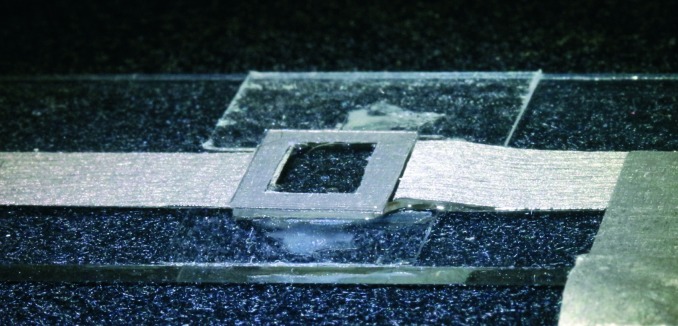
Photograph of the probe chamber (bottom view), with electrical contacts made out of conductive adhesive tape, and mounted on a microscope slide (outer dimensions probe chamber 10 mm × 10 mm).

At the heart of the setup is the manipulation chamber, as depicted in Fig. 1 and 2. It consists of a fluid layer, sandwiched between a transducer and a glass cover slip, which acts as a reflector for acoustic waves travelling along the axial (vertical) direction. Double-sided adhesive tape acts as a spacer and seals the manipulation chamber. Typical layer thicknesses and material parameters are given later in Table 1. We also successfully trialled rectangular glass capillaries (Vitrocom W3520, wall thickness 200 μm, fluid layer thickness 200 μm, and width 4 mm) as a disposable, easy to exchange probe chamber. Nevertheless, in this work, we restrict ourselves to the basic design of Fig. 1, which we found to be more flexible with better performance and uniformity.

**Table 1:** Model parameters used for the simulations. The values marked with † are deduced from a comparison of model calculations with experimental data, the others are nominal values taken from the literature. The material quality factor describes the damping and radiation losses within a layer, *i.e.* the propagation of a harmonic wave 
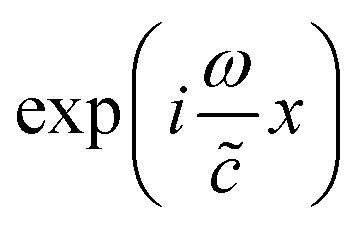
 is described by a complex sound velocity 
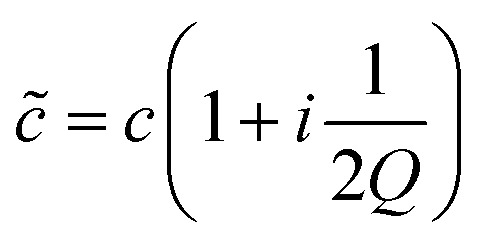
 ^17^

Layer	Material	Thickness *d* (μm)	Speed of sound *c* (m s^–1^)	Density *ρ* (kg m^–3^)	Material quality factor *Q*
Immersion oil	Immersol 518 N	*	1300	1094	1^†^
Mounting slide	Glass	1032^†^	6000	2500	300^†^
Immersion oil	Immersol 518 N	77^†^	1300	1093	80^†^
Transducer	LiNbO_3_	500	7340^†^	4650	100^†^
Fluid	D_2_O	125^†^	1480	1050	100^†^
Cover slip	Glass	170	6000	2700	100^†^
Immersion oil *n* = 1.33	Immersol W 2010	100 to 300	850^†^	1800	40^†^
Objective lens	Glass	*	5000	3000	1^†^

#### 2.1.1 Design considerations

The requirements for optical imaging, trapping and force measurement pose some constraints on design. First, for focussing the trapping light and for imaging, we employ a high-NA water immersion objective lens (Olympus UPlanSApo 60× W, NA 1.2). This lens needs a cover glass with a thickness of 130 μm to 200 μm. Second, our force measurement method^20^ requires the collection of most of the trapping light. To achieve this, we use a transparent piezo-electric transducer and a high-NA oil immersion condenser (see sec. 2.2 and 2.4).

In order to obtain strong acoustic forces, our probe chamber design pays attention to the following guidelines:^22^ one aim is to drive the transducer close to its fundamental thickness resonance (thickness *λ*/2), exciting a resonantly enhanced standing wave in the fluid chamber, for which we also choose a thickness of approximately *λ*/2. We stay with standard cover slips with a thickness of 170 μm, as this is non-critical for the acoustic trapping.^22^ We have no coupling layer between the transducer and the fluid.

Due to the large differences in the acoustic impedance *Z* = *ρc* between transducer, fluid and cover slip (by a factor of >10, see Table 1), strong reflections of acoustic waves occur at the interface, and a resonantly enhanced standing wave will emerge in the fluid chamber.^5^ From these considerations, we expect for the fundamental *λ*/2 resonance a sinusoidal force profile with a stable trapping position in the center of the fluid layer, and approximately zero force at the boundaries.^6^

The probe chamber assembly (transducer, spacer and cover glass) is mounted on a microscope slide (thickness 1 mm) with the help of adhesive tape stripes and a drop of superglue to increase the bond, see Fig. 2. The gaps between transducer and mounting slide and between mounting slide and condenser lens are filled with immersion oil.

### 2.2 Transparent piezo-transducer

The transparent transducer employed,^21,23^ which was made out of lithium niobate (LiNbO_3_, 36° Y-cut, 10 mm × 10 mm × 0.5 mm, manufactured by Roditi, UK), has a transparent indium tin oxide (ITO) conductive coating (Diamond Coatings, UK) with a sheet resistance of approximately 10 Ω.

Electrical contact is made by a conductive adhesive tape (Hi-Bond Tapes HB350, RS 832-6366), attached to the transducer at opposing edges, see Fig. 2. In order to ensure reliable electrical contact, we partly remove the glue layer and instead apply silver loaded paint (RS 186-3593).

### 2.3 Low cost signal source and electrical impedance measurements

As an electrical signal source and for measuring the electrical impedance of the transducer we use a Red Pitaya V1.1 device. It offers two analog outputs and inputs, digitally sampled at 125 MHz rate. Both output channels are summed and amplified by a power amplifier (ADA4870 from Analog Devices on evaluation board, RS 836-8714), to which the transducer is connected by a pair of 20 cm long wires. The voltage at the power amplifier output and across a 10 Ω shunt resistor (in series with the amplifier output) is fed back to the analog inputs of the Red Pitaya device, providing information about the (complex) electrical impedance of the transducer (including the contribution of the wires). Signal generation, data acquisition and subsequent analysis is controlled by a custom Python program, running on the embedded processor of the Red Pitaya device under a Debian Linux operating system. This setup provides a versatile and powerful solution for signal generation and impedance measurement at low cost (approx. € 300).

### 2.4 Optical setup for imaging and optical trapping

Our experimental setup for realizing simultaneous acoustic and optical manipulation is comprised of an inverted microscope (Zeiss Axioscope 135) with a couple of additions for optical manipulation. As already stated above in sec. 2.1, we use a high-NA objective lens (Olympus UPlanSApo 60× W, NA 1.2) both for imaging and focussing the trapping light. Designed for use with water immersion, this lens provides good imaging and beam quality even when focussing deep into the fluid layer. For the immersion medium we prefer to use oil with an water-like refractive index of *n* = 1.33 (Immersol W, Zeiss) instead of water to avoid degradations due to evaporation.

A high-NA oil condenser (Olympus U-AAC, NA 1.4) is used for force detection and illumination, as described in more detailed in the following sections.

#### 2.4.1 Imaging and illumination

For a spatially incoherent illumination of the sample we use a green LED, evenly filling the back aperture of the condensor. Alternatively for a coherent, plane wave illumination we focus the beam of a fiber coupled diode laser at 640 nm at the back aperture. In this configuration the presence of particles far outside the focal plane is observable. We also employ it for inline holography to determine the particle size.^24^ For image acquisition we use a digital camera at the camera port of the microscope (mvBlueFOX3 BF3-2024G from Matrix-Vision with a Sony IMX174 sensor).

#### 2.4.2 Holographic optical tweezers

For optical trapping we introduce a laser beam, derived from a fiber laser at 1064 nm (PYL-10-1064-LP, IPG Laser) into the optical path of the microscope, with typically about 70 mW going into the objective lens.

With the help of a liquid crystal based spatial light modulator (SLM, model P512-1064 from Boulder Nonlinear Systems), which is imaged on to the back focal plane of the objective lens with a 4f-setup, we holographically control the focus position, which gives a radial and axial range of about 100 μm. The SLM pattern is computer controlled, a custom software provides real-time pattern calculation for an interactive or automated operation of the optical tweezers.

#### 2.4.3 Optical force detection

For measuring the optical force exerted on a trapped particle we image the intensity distribution of the recollected trapping light in the back focal plane of the condenser onto a digital camera (mvBlueFOX3 BF3-2024G, Matrix-Vision) equipped with a *f* = 50 mm lens, Tamron 23FM50SP, and an attenuator with 0.1% transmission), revealing the angular momentum distribution of the outgoing trapping light in the forward direction.^20^ From the images, acquired at a rate of 200 Hz, we calculate the exerted force in all directions.

This direct force measurement method is robust and essentially calibration free.^19,20,25^ Unlike with conventional back focal plane interferometry, large forces close to the maximum trapping (escape) force can be reliably measured, since it does not rely on a linear relationship between detector signal and force. This is advantageous for improving the signal-to-noise ratio and favours productive measurements due to shorter averaging periods and omission of recalibration steps. We confirmed the validity of this approach by applying a known drag force, translating a trapped microsphere at a controlled speed.

### 2.5 Test samples

For the detailed measurements we use silica microspheres with a diameter of approximately 3 μm (Whitehouse Scientific). Compared to polystyrene microspheres, commonly used for optical trapping, they show less backscattering of light due to their lower refractive index, enabling accurate direct axial force measurements^20^ even without measuring the light scattered in the backward direction. As a precautionary measure, the particles are prepared in heavy water (D_2_O) to reduce absorption of the trapping light at 1064 nm and to offset possible thermal effects (*e.g.* convective fluid flows, which could induce drag forces).

## 3 Results and discussion

In the following we present the experimental results we have obtained for the combination of acoustic trapping with holographic optical tweezers. In particular we present a detailed characterization of the acoustic properties of the probe chamber with the help of force measurements using an optically trapped test particle. This provides key information that is otherwise difficult to access. To give a consistent picture, the major part of the results presented here have been determined with a single probe chamber.

### 3.1 Combined acoustic and optical manipulation of large specimens

Before we present detailed investigations, we start with a simple demonstration of the capabilities of combined acoustic and optical trapping.^26^ One strength of acoustic trapping is that the force on small particles (in our case <100 μm and hence smaller than the acoustic wavelength being employed) scales with the particle volume.^6^ In consequence, with acoustic forces particles can be trapped which are much larger than it would be possible with optical forces alone. Furthermore, acoustic trapping has few requirements on the physical properties of the particles: nearly any particle can be trapped, either in the pressure nodes or anti-nodes, depending on the acoustic contrast factor.^6^ Conversely, optical trapping has stricter requirements (limits on size, shape and refractive index, low light absorption or scattering), and stable axial trapping is notoriously more difficult to achieve, especially with single beam optical tweezers. Furthermore, the maximum optical force is ultimately limited by the momentum carried by light, (*F*_max_ < 2*P*/*c*) and thus by the maximum laser power *P* that is available or compatible with maintaining sample viability. In practice, with 100 mW of light a maximum force of roughly 10 pN is typically realized.^3^

However, in combination acoustic and optical manipulation provide the “best of two worlds”, *i.e.* they unite the possibilities provided by largely different wavelengths (∼1 μm *versus* ∼100 μm), and enable the handling of particles which would not be possible with the individual methods alone, *e.g.*, large particles are levitated and confined within a plane by acoustic forces and precisely manipulated by optical forces.

**Fig. 3:**
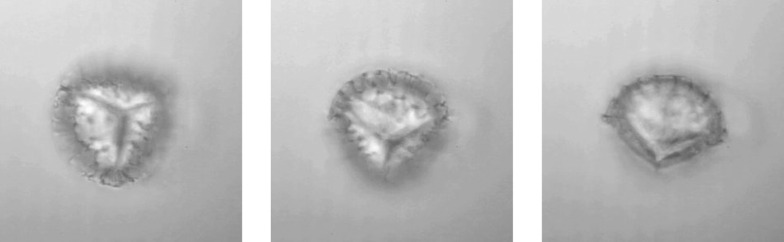
*Lycopodium* spore, levitated by acoustic forces and held in place by the optical trap, continuously rotates (image taken from movie, 0.5 s time steps).

To demonstrate this we trap *Lycopodium* spores with typical sizes of 30 μm to 35 μm (Fig. 3). When switching on the ultrasound, the spores are detached from the bottom surface and levitated by acoustic forces. With help of the optical trap it is possible to drag the particles within the nodal plane. In addition to our previous work^26^ we now have the additional option of high-resolution imaging. We also observe that the levitated spores continuously rotate around an axis within the nodal plane, offering views from different angles and, *e.g.*, permitting volumetric imaging based on tomography.

### 3.2 Accessible force range

**Fig. 4:**
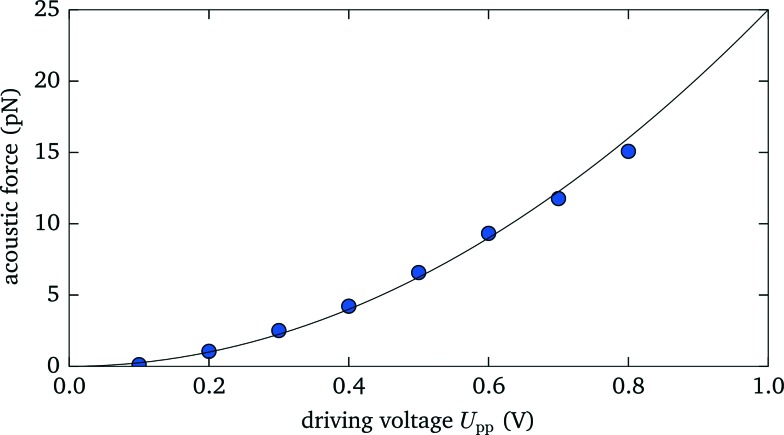
The measured acoustic force strength acting on a 3.5 μm diameter silica microsphere scales quadratically with the applied voltage.

For many applications the maximum attainable force plays an important role, *e.g.*, for acoustic force spectroscopy of single DNA strands^1^ one wants to achieve forces of ∼100 pN, large enough to overstretch DNA. Here we show that with our setup we are able to create significant acoustic forces, exceeding the range typically accessible with optical tweezers. Within a 105 μm thick fluid layer we excite the fundamental resonance at 7.2 MHz. We optically trap a 3.5 μm silica microsphere at an axial position of 70 μm, where the force reaches its peak value (see also sec. 3.5), and measure the exerted force for different driving voltages *U*_p–p_ (given as peak-to-peak value). As expected, we observe a quadratic scaling of the acoustic force with increasing voltage,^6^ as shown in Fig. 4. Above *U*_p–p_ > 0.8 V the maximum optical force of about 15 pN is exceeded and the particle is expelled from the optical trap. Extrapolating the acoustic force at the maximum driving voltage of *U*_p–p_ = 20 V, which we are able to reach with our setup, would yield an acoustic force of ∼10 000 pN, several orders of magnitude larger than the attainable optical force.

### 3.3 Characterization of the probe chamber: acoustic and electric response

In this and the following sections we analyze in detail the properties of a probe chamber with a 125 μm thick fluid layer. For demonstration purpose this design intentionally deviates from the rules for optimized force strength to instead obtain a behaviour with well separated resonances, which is easier to interpret.

**Fig. 5:**
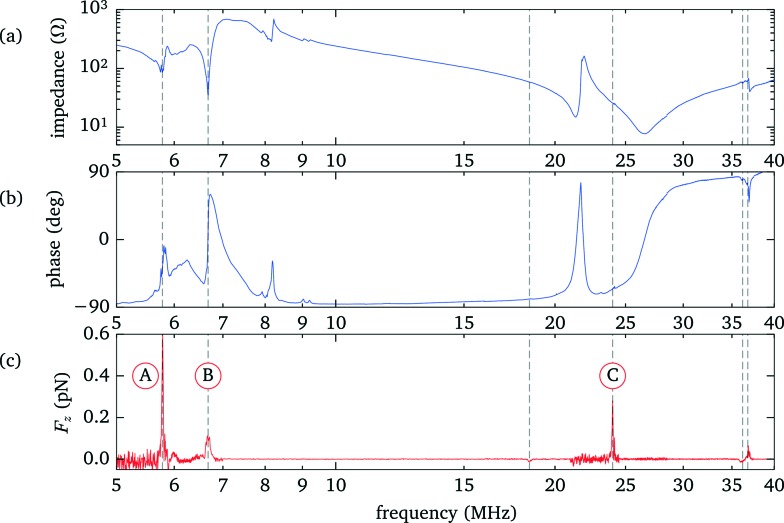
Electrical response and acoustic force when scanning the driving frequency. (a) Magnitude and (b) phase of the electrical impedance of the transducer. (c) Acoustic forces acting on a 3 μm silica bead. To make weak resonances more easily recognizable, larger driving voltages were used in regions containing only weak resonances. Shown is the rescaled force *F*/*U* ^2^, leading to an uneven noise floor level.

To identify the resonances within the fluid we perform a frequency scan while recording the force acting on an optically trapped silica bead placed at a position about 3/4 of the fluid layer thickness. Simultaneously we measure the electrical impedance of the transducer. The results are presented in Fig. 5. The strongest force is observed at about 5.8 MHz (marker A) and corresponds to the fundamental *λ*/2 mode of the fluid layer, as shown in more detail in sec. 3.5. Another weaker peak at 6.7 MHz (marker B) is related to the fundamental resonance of the transducer. Both features are also clearly recognizable in the electrical response. The third prominent force peak at 23.9 MHz (marker C), now hardly visible as a small peak in the phase, is due to a higher mode in the fluid layer.

#### 3.3.1 Comparison with model calculations

**Fig. 6:**
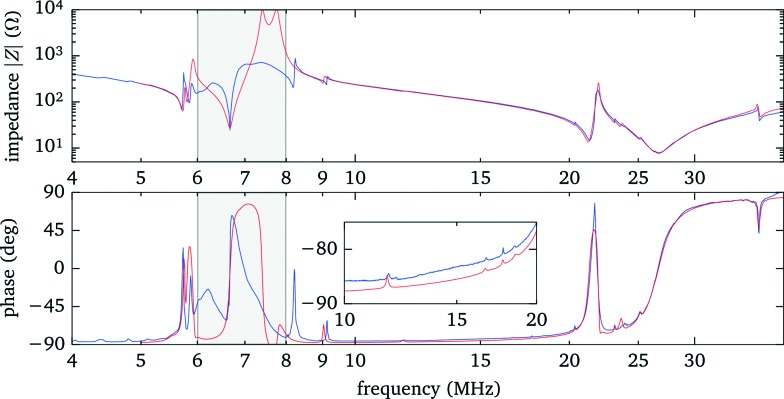
Comparison between measured electrical impedance data (blue line) and 1D model calculations (red line). In the range from 6 MHz to 8 MHz, close to the fundamental resonance of the bare transducer, the simulations, neglecting *e.g.* the border area of the transducer covered by the spacer, only roughly match the data, whereas further away the data are well described (see inset). For this data there was no immersion oil added between mounting slide and condenser, making small features more prominent.

To get proper insight we compare the experimental results, in particular the electrical response data, with 1D model calculations (ESI†).^22^ In addition to the data shown in Fig. 5 we have analyzed electrical response data for configurations where the gaps between the transducer and the mounting slide, as well as between the mounting slide and the condenser, are not yet filled with immersion oil. From this we obtain values for the model parameters, in particular the layer thicknesses, given in Table 1. Overall we find a good agreement between model calculations and experimental data, see Fig. 6.

Using the electrical response data has the advantage that individual resonances in any single layer are observable, albeit often only as weak features, such as small peaks in the phase. This unambiguously determines, *e.g.*, the parameters of the mounting slide and the gap between it and the transducer. (We note that only the product *dc* of thickness *d* and speed-of-sound *c* can be deduced from the comparison of experiments with model calculations, therefore the individual values are not well defined in cases where both *d* and *c* are only roughly known.) However, coupled resonances in different layers with similar frequencies are more difficult to model. In particular, in the region between 6 MHz to 9 MHz, close to the fundamental resonance of the bare transducer, we see deviations between experiment and simulation. We attribute this, at least in part, to the fact that our 1D model does not include the contribution of the spacer and the electrode contacts around the fluid chamber. However, we take into account that only about 1/3 of the transducer is covered with the fluid by accordingly scaling the total acoustic impedance seen by the transducer. The other areas covered by the spacer and electrodes add in parallel an (unknown) acoustic load and damping to the transducer, thus changing the electrical response, especially close to the fundamental of the bare transducer. However, these areas are not directly coupled to the fluid layer and therefore have a limited effect on the acoustic resonances within the fluid, thus we simply neglect them.

In our model we also include the series inductance of 0.53 μH of the separate wires connecting the probe chamber electrodes to the power amplifier, which together with the capacitance of the transducer and the sheet resistance of the transducer electrodes (modelled as a series resistance of 7.5 Ω) leads to the prominent electrical series resonance at 27 MHz with the 180° phase shift.

### 3.4 Influence of coupling to objective and condenser lens

The use of an immersion objective lens, required for high-NA imaging and optical trapping, adds additional layers that are acoustically coupled to the probe chamber. When moving the objective lens to focus at different positions within the fluid layer, the thickness of the immersion layer changes and correspondingly the acoustic resonances are affected. In this section we study in detail how the position of the objective influences the acoustic forces and how to cope with experimental issues arising due to this interaction.

**Fig. 7:**
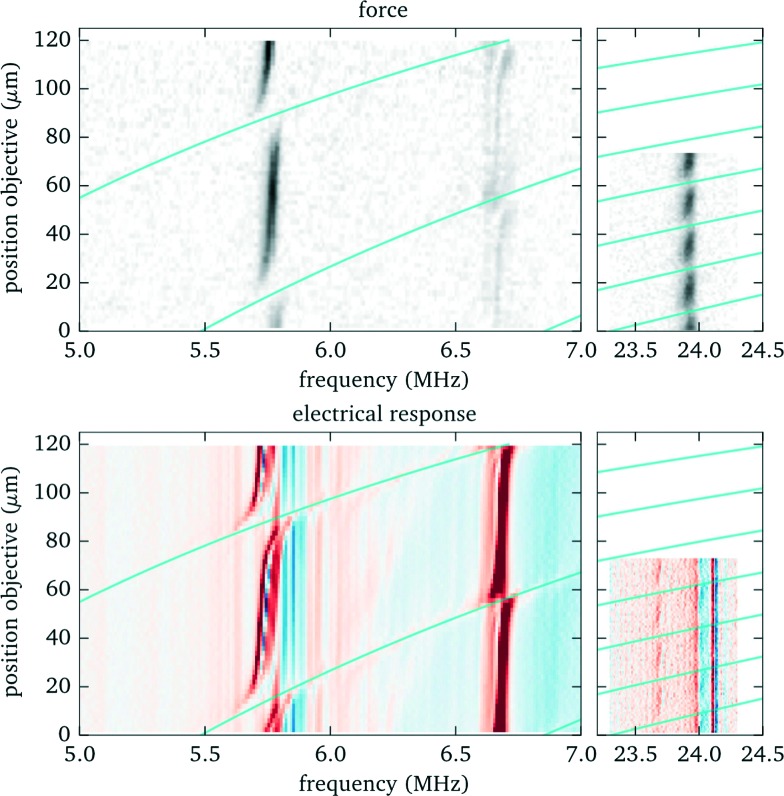
Influence of objective position on acoustic resonances. Top: Acoustic force (force strength depending on frequency and objective position encoded as grayscale value). Bottom: Electrical response (to enhance small features the derivative of the phase is shown as a colour image). The solid lines indicate the conditions, based on eqn (1), where an acoustic resonance within the immersion layer (between cover glass and objective lens) occurs. Here we define the origin for the objective position (*z* = 0) when its focal plane coincides with the interface between cover slip (upper surface) and fluid, corresponding to a gap of 310 μm between objective lens and (bottom) cover glass surface.

To experimentally characterize the behaviour we perform force measurements for a range of axial positions of the objective lens covering the full height of the fluid chamber, and scanning the frequency across the most prominent resonances. We keep the position of the bead within the probe chamber fixed at 90 μm above the bottom, compensating with the holographic beam shaping of the optical tweezers for the axial displacement of the focal plane when translating the objective lens. We observe (see Fig. 7) that for some positions of the objective the acoustic resonance within the fluid chamber vanishes (*e.g.* for the resonance near to 5.8 MHz this happens close to *z* = 15 μm and *z* = 90 μm). Additionally, the resonance frequency shows a periodic shift depending on the objective position. For applications one needs to take these effects into account. In the remaining part of this section we discuss the source of this behaviour and how to mitigate its impact.

#### Simple relationship to estimate the location of the force gaps

**Fig. 8:**
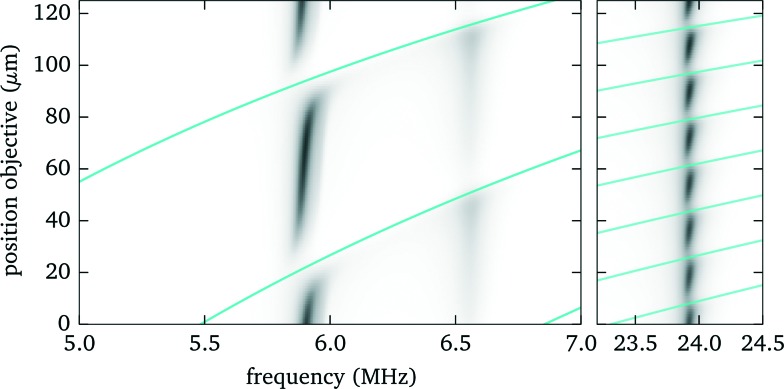
Simulation result for the dependence of the acoustic force on the objective position and frequency, similar to Fig. 7.

More detailed investigations and model calculations (see Fig. 8) reveal that this behavior originates from acoustic resonances within the immersion layer between objective and cover glass. Whenever a resonance frequency of the immersion layer nearly coincides with one of the fluid, the resonances couple to each other and we observe resonance splitting (visible as “avoided crossings” in Fig. 7) accompanied by a reduced strength due to the larger damping in the immersion layer. Resonances within the immersion layer occur whenever its thickness is an integer multiple of half the wavelength *λ*/2, *i.e.* at frequencies1
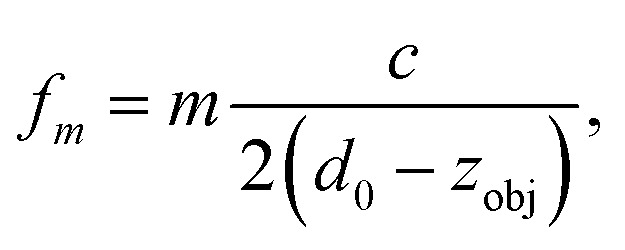
where *m* denotes an integer number, *z*_obj_ the objective position, and *d*_0_ = 310 μm the immersion layer thickness when focussing at the inner cover glass surface (*z*_obj_ = 0), *i.e.*, the working distance of the objective lens. These resonances are indicated in Fig. 7 and 8 as solid lines. It is straightforward to determine in advance the positions of the objective lens where the acoustic force vanishes, even for a different probe chamber design, *e.g.* with changed fluid layer thickness. Essentially only the resonance frequencies need to be measured or estimated from model calculations.

#### Strategies to avoid force gaps

According to eqn (1) one even has several options to control at which lens positions the resonance crossings take place:

• by exchanging the immersion medium to control the speed of sound, *e.g.* water against immersion oil (with refractive index like water) or *vice versa*,

• by using a cover slip with different thickness to change the immersion layer thickness (requires an objective lens with a correction collar),

• by changing the thickness of the fluid layer to change its resonance frequency.

By taking one or more of these measures one can avoid situations where the force gaps coincide with the objective position needed to observe trapped particles in the acoustic node. However, higher modes are more susceptible (see Fig. 7 and 8), since more resonance crossings occur for the same shift in objective position.

#### Strategies to compensate for resonance frequency shifts

Outside the resonance crossings the acoustic trapping is only moderately affected by changing the objective lens position. Although not strictly required, it is advisable to adjust the driving frequency to stay right on resonance and assure optimum acoustic forces. An important observation for this purpose is that the fluid resonances are also observable in the electrical response of the transducer (see Fig. 7), which can be measured with little effort.

#### Influence of the condenser lens position

In brief, we observe both experimentally, and by simulation, that the position of the condensor lens hardly affects the resonances within the fluid layer. We presume that the additional layers between fluid layer and condensor immersion layer lead to a reduced acoustic coupling. In general, there is no need to adjust the condenser position during experiments.

### 3.5 Measurement of force profiles

**Fig. 9:**
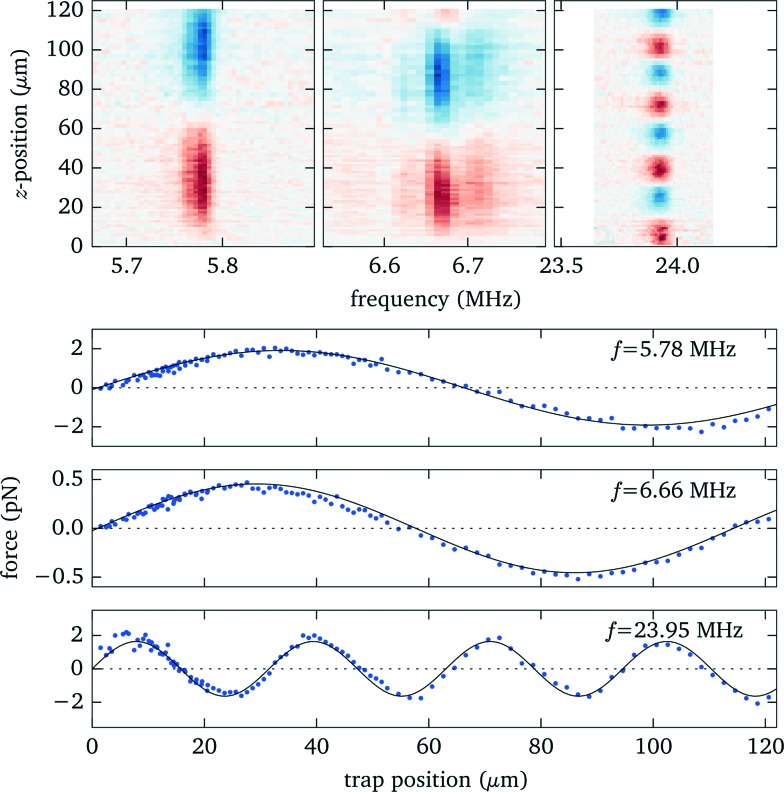
Measured force profiles for the three most prominent acoustic resonances. The solid black lines in the lower panel are sinusoidal fits to the experimental data.

Knowing the force profiles, in particular the position of the nodal planes, is important for acoustic trapping. In this section we present measurements of the axial force profiles for several resonances, see Fig. 9. For this we keep the position of the objective fixed at 70 μm, avoiding resonance crossings for all of the resonances studied in Fig. 7, and move the trap position by changing the pattern used for the holographic optical tweezers. For the low frequency modes near 6 MHz we observe sinusoidal force profiles with a single stable trapping position roughly in the center of fluid layer, whereas for the strong high frequency mode close to 24 MHz there exist four nodal planes with stable trapping. Furthermore, we find for this design non-zero acoustic forces at the upper boundary, which can be used to either detach particles or press them against the surface. Such forces near the surface have also been used to probe DNA strands tethered between the surface and micro-spheres.^1^

**Fig. 10:**
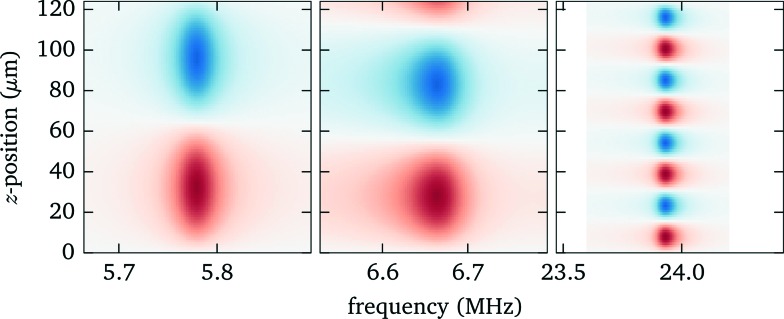
Force profiles from model calculations.

The corresponding model calculations for the force profiles (see Fig. 10) agree well on the *location* of the nodes. However, the simulations, which employ frequency independent material quality factors, cannot properly reproduce the strength and width of the resonances, in particular the low frequency modes are in reality narrower and show some substructure. It appears that the damping, which influences the resonance width, increases at higher frequencies (as to be expected qualitatively from Stokes law for viscous sound attenuation in a fluid).

### 3.6 Force profile shaping

In this section we demonstrate how to engineer the force profile by simultaneously applying two excitation frequencies, and we give a specific example where we have experimentally mapped out the resulting more complex shape. While the force profile for a specific single resonance exhibits a sinusoidal shape, simultaneously exciting several modes (or alternatively quickly switching between several frequencies^27^) gives more freedom to realize modified force profiles.

**Fig. 11:**
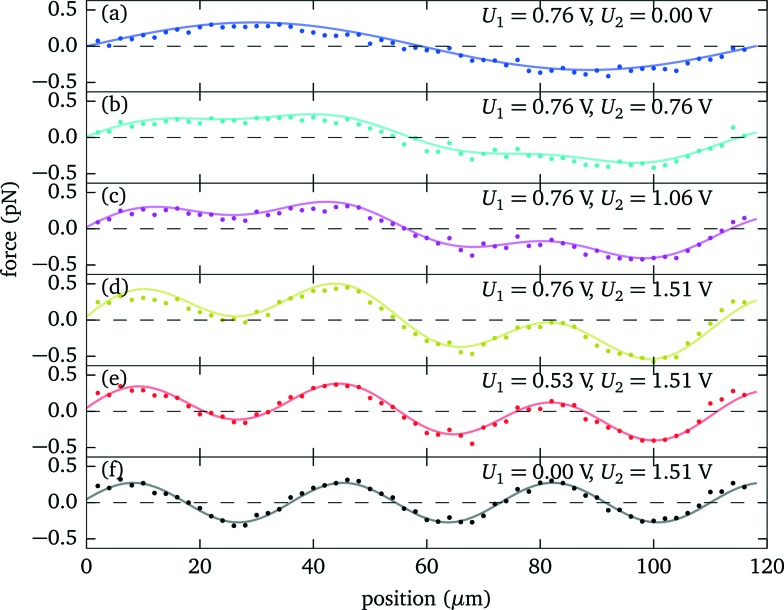
Force profile engineering by simultaneously exciting two resonances at 6.7 MHz and 21.7 MHz of a 118 μm thick fluid layer. (a)–(f) Depending on the relative strength of the individual amplitudes *U*_1_ and *U*_2_, different force profile shapes can be realized. For the individual modes (a) and (f) the force profiles are well described by a sinusoidal shape (solid lines), determining *F*_1_(*z*) and *F*_2_(*z*) of eqn (2).

According to the quadratic scaling of the force with the driving voltage^6^ (see also sec. 3.2), the total force is given as the weighted sum of the individual contributions, which for two different modes can be expressed as2
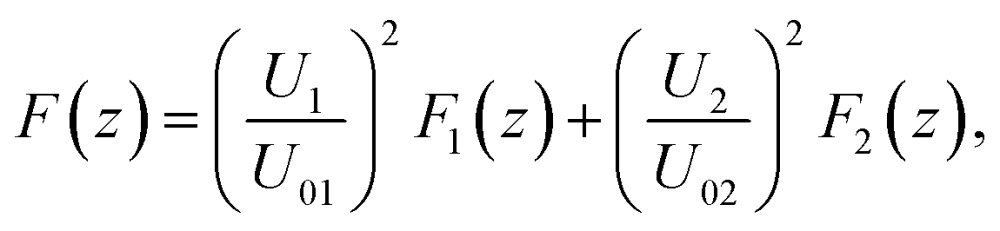
where *F*_*i*_(*z*) denotes the individual force profile for a driving voltage of *U*_0*i*_. This is demonstrated in Fig. 11. Here we use two resonances at 6.7 MHz and 21.7 MHz of a different probe chamber (fluid layer thickness 118 μm) with one and three stable trapping positions, respectively. Each of the individual force profiles is well described by a sinusoidal shape with a period of 59 μm or 18.5 μm, respectively. When changing the individual amplitudes *U*_1_ and *U*_2_, the observed force agrees well with the behaviour as expected from eqn (2). This refined control provides new possibilities, such as moving the trapping positions, realizing a more uniform force over an extended range (*e.g.* at positions 10 μm to 45 μm in Fig. 11b), or steepening the force gradient near the single stable trapping position (see Fig. 11c).

### 3.7 Lateral variations of acoustic forces

For massive parallelization of acoustic trapping it is important to achieve uniform forces across the lateral extent of the probe chamber. Thickness variations of the layers or boundary effects can negatively affect the uniformity. By locally probing the acoustic forces with our method we are able to reveal possible inhomogeneities.

**Fig. 12:**
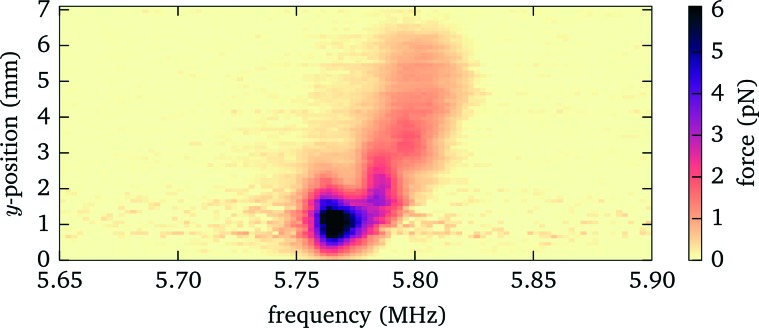
Acoustic force strength *versus* driving frequency and lateral position when moving the probe chamber laterally.

For our probe chamber we observe only insignificant variations of the acoustic forces within the field of view (diameter about 300 μm). However, on a larger scale, when we move the probe chamber laterally to assess the full fluid chamber (about 7 mm), we detect changes in the acoustic force, see Fig. 12. Both the frequency, where the maximum force is observed, as well as the peak force are affected. From the observed frequency shift we deduce that the thickness variation of the fluid layer is less than 1 μm, if that is the only cause. The variation in force strength and frequency could also be explained by a changing thickness of the immersion layer between objective lens and cover glass, caused by a vertical oscillation in our microscope stage when translating the probe chamber horizontally. As shown in Fig. 7, at the position of about *z* = 70 μm used for this measurement, the force strength (and resonance frequency) is susceptible to changes in the distance between objective lens and probe chamber.

## 4 Conclusions

The combination of optical tweezers with acoustic trapping, based on a layered resonator design, unites parallel handling of a large number of particles with precise control on the single particle level. The use of a transparent piezo transducer made out of LiNbO_3_ provides good optical access and facilitates straightforward integration with little effort into existing optical tweezers setups. This enables robust, calibration free 3D force measurements based on the direct observation of the momentum transfer from the trapping beam to the particle, giving detailed, spatially resolved information about acoustic forces within the probe chamber. Furthermore, as this force measurement method is independent of the particle shape, it enables the characterization of acoustic forces on particles with irregular shapes, such as cells, for which, as an example, conventional methods based on drag forces are not applicable. In combination with complementing measurements of the electrical response of the transducer, we are able to determine the model parameters of a 1D simulation of the acoustic behaviour, useful for optimizing control and design of acoustic trapping.

In our setup the acoustic forces act entirely in the (vertical) direction normal to the planar resonator, nevertheless our approach is also applicable and useful to characterize probe chamber designs with transverse forces, *e.g.*, in microfluidic devices with narrow channels or transverse acoustic resonators. Also, our method allows for a detailed quantitative mapping of the 3D force field near a particle due to secondary acoustic radiation forces,^17^ which is impractical with conventional methods such as particle tracking.

Our results are relevant for combining acoustic trapping and imaging with the high-NA lenses needed, *e.g.*, to attain high resolution and high sensitivity. With some precaution any adverse influence of the high-NA imaging lens on the acoustic forcing can be avoided. For a convenient operation of the setup we envisage that continuously monitoring the electrical impedance of the transducer, which we already realized with our custom signal source, will allow us to automatically adapt the settings, *e.g.* the frequency to compensate for shifts in resonance frequency.

An important application, where such a combination of acoustic manipulation and imaging is desirable, is acoustic force spectroscopy (AFS),^1^ where DNA strands, tethered between microspheres and a surface, are stretched by acoustic forces in a planar resonator. Combining it with high-NA fluorescence imaging would allow the observation of the dynamic binding of (single) proteins to DNA under tension by fluorescence microscopy^28^ in a highly parallel manner. Additionally, the demonstrated force profile engineering would provide an improved performance for this application, such as a flat force profile.

The rather strong acoustic forces, even increasing with particle size, opens the possibility to handle not only cells, but larger entities such as cell clusters, large micro-organisms or whole organisms. Our setup provides possibilities, *e.g.*, to levitate or detach them from surface, investigate them by high resolution imaging, and sort or transport them by optical forces.
