# Supplementary material for: Acoustic force mapping in a hybrid acoustic-optical micromanipulation device supporting high resolution optical imaging
Source: Lab Chip. 2016 Mar 17;16(8):1523–32. doi: 10.1039/c6lc00182c (PMC5058352; doi:10.1039/c6lc00182c)
Supplement: Supplementary file 1 [file LC-016-C6LC00182C-s001.pdf]

Electronic Supplementary Material (ESI) for Lab on a Chip.
This journal is © The Royal Society of Chemistry 2016

SUPPLEMENTARY INFORMATION

Acoustic force mapping in a hybrid acoustic-optical micromanipulation device supporting high resolution optical imaging

1D model for a piezoelectric transducer with layered acoustic resonator

In this Supplementary Information we summarize the 1D model which we use to simulate the response of our device, which consists of a piezoelectric transducer made out of $LiNbO_3$ coupled to a stack of layers on top and bottom. We closely follow the standard approach for modelling ultrasound transducers, an in-depth description for which is contained e.g. in [1] and [2].

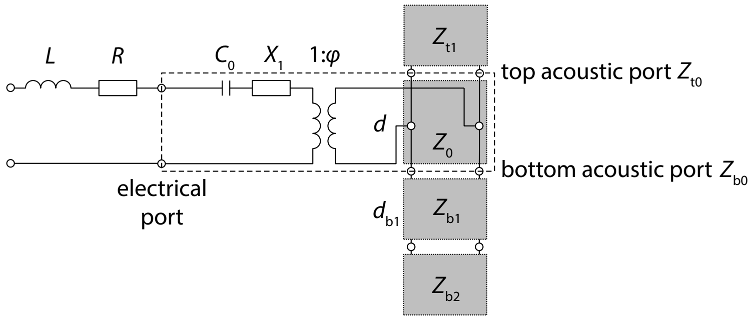

Figure 1: Equivalent circuit for a layered piezoelectric acoustic resonator. The KLM-model for the bare transducer is delimited by the dashed line. Additionally shown are a top and two bottom layers, plus representative elements L and R describing the inductance and resistance, respectively, of the wires and electrodes attached to the transducer.

KLM-model for piezoelectric transducer

The transducer is modelled by an equivalent electrical circuit [3], as depicted in Fig. 1. In the KLM-model the transducer is described by representative elements, coupled to an acoustic

label	description	value
A	transducer area	$100\,\mathrm{mm}^2$
d	thickness transducer	$500\,\mu\mathrm{m}$
ρ	density transducer	$4650\,\mathrm{kg\,m}^{-3}$
c	speed of sound†	$7340\,\mathrm{m\,s}^{-1}$
ϵ_{33}/ϵ_0	dielectric permittivity†	39
h_{33}	piezoelectric constant†	$12.9\times10^9\,\mathrm{V\,m}^{-1}$
$\tan\delta_E$	dielectric loss factor	0.003
Q	material quality factor	100
L	inductivity wires†	$0.5\,\mu\mathrm{H}$
R	resistance electrodes†	$7.5\,\Omega$

Table 1: Values for material parameters of the $LiNbO_3$ transducer (36° Y-cut). The entries marked by † have been obtained by comparison of model calculations with measurements taken with a bare transducer.

transmission line. For excitation with frequency $\omega = 2\pi f$ their values are given by

$$C_0 = \epsilon'_{33}\frac{A}{d},$$
$$X_1 = jZ_0M^2\sin(kd), \tag{1}$$
$$\varphi = \frac{1}{2M\sin(kd/2)},$$

with $M = \frac{h_{33}}{\omega Z_0}$, the acoustic impedance $Z_0 = \rho c' A$, and the wave number $k = \frac{\omega}{c'}$. Losses are included by introducing complex material parameters

$$\epsilon'_{33} = \epsilon_{33}(1 - j\tan\delta_E), \tag{2}$$
$$c' = c\left(1 - \frac{j}{2Q}\right). \tag{3}$$

The values of the material parameters for the transducer are given in Table 1.

Acoustic impedance of layer stack

The next step in the model calculations is to determine the acoustic impedance presented by the layered stack to the top and bottom surface of the transducer. For the sake of simplicity we only present the calculation of the acoustic impedance of a layered stack for the specific case of a single intermediate layer, attached to the bottom surface of the transducer.

This layer and its material is described by the area A_{b1}, the thickness d_{b1}, the speed of sound c_{b1}, the density ρ_{b1}, and the quality factor Q_{b1}. From these parameters the acoustic impedance $Z_{b1} = A_{b1}\rho_{b1}c_{b1}$ and (complex) wave number $k_{b1} = \frac{\omega}{c_{b1}(1-\frac{j}{2Q_{b1}})}$ are determined. We assume that this intermediate layer 1 is connected to a layer 2 (infinitely thick) with acoustic

impedance Z_{b2}. Then the acoustic impedance exposed to the transducer is given by

$$Z_{b0} = Z_{b1} \frac{Z_{b2} + jZ_{b1} \tan(k_{b1} d_{b1})}{Z_{b1} + jZ_{b2} \tan(k_{b1} d_{b1})}. \tag{4}$$

If more than one intermediate layer is present, one has to repeatedly apply Eq. (4), starting from the most distant layer.

Calculation of electrical impedance

The (total) acoustic impedances Z_{to} and Z_{bo} presented to the transducer at the top and bottom surface are converted to impedances at the center tap

$$Z_t = Z_0 \frac{Z_{to} + jZ_0 \tan(kd/2)}{Z_0 + jZ_{to} \tan(kd/2)}, \tag{5}$$

$$Z_b = Z_0 \frac{Z_{bo} + jZ_0 \tan(kd/2)}{Z_0 + jZ_{bo} \tan(kd/2)}, \tag{6}$$

which are connected in parallel to yield

$$Z_{\parallel} = \frac{Z_t Z_b}{Z_t + Z_b}. \tag{7}$$

Finally, from this the electrical impedance presented to the signal source is obtained by

$$Z_{in} = \frac{1}{j\omega C_0} + X_1 + \frac{Z_{\parallel}}{\varphi^2} + j\omega L + R, \tag{8}$$

where we also included contributions from the leads (inductance L) and the transparent electrodes (resistance R).

Calculation of acoustic pressure within layer stack

Furthermore we want to know the acoustic forces generated by the transducer. If we apply a voltage V_{in} to the input leads, then the force at the (bottom) surface is given by

$$F_{bo} = \frac{V_{in} Z_{\parallel}}{Z_{in} \varphi} \frac{Z_{bo}}{Z_{bo} \cos(kd/2) + Z_0 j \sin(kd/2)}. \tag{9}$$

For the calculation of the acoustic radiation forces acting on a particle we refer to [4, 5].

References

[1] G. S. Kino, *Acoustic waves: devices, imaging, and analog signal processing*, Prentice-Hall, Englewood Cliffs, N.J., 1987.

[2] M. Gröschl, *Acta Acustica united with Acustica*, 1998, **84**, 432–447.

[3] R. Krimholtz, D. A. Leedom and G. L. Matthaei, *Electronics Letters*, 1970, **6**, 398–399.

[4] M. Hill, Y. Shen and J. J. Hawkes, *Ultrasonics*, 2002, **40**, 385–392.

[5] H. Bruus, *Lab on a Chip*, 2012, **12**, 1014.
